# Transcriptome and Network Analyses Reveal the Gene Set Involved in PST Accumulation and Responses to Toxic *Alexandrium minutum* Exposure in the Gills of *Chlamys farreri*

**DOI:** 10.3390/ijms23147912

**Published:** 2022-07-18

**Authors:** Yujue Wang, Moli Li, Jiarun Lou, Xiaogang Xun, Lirong Chang, Yangrui Wang, Quanqi Zhang, Longfei Lu, Huizhen Wang, Jingjie Hu, Zhenmin Bao, Xiaoli Hu

**Affiliations:** 1 MOE Key Laboratory of Marine Genetics and Breeding, College of Marine Life Sciences, Ocean University of China, Qingdao 266003, China; wangyujue1236@126.com (Y.W.); lml940520@163.com (M.L.); ljr9481@163.com (J.L.); xxgsemail@126.com (X.X.); changlr@163.com (L.C.); wangyangrui@stu.ouc.edu.cn (Y.W.); qzhang@ouc.edu.cn (Q.Z.); hujingjie@ouc.edu.cn (J.H.); zmbao@ouc.edu.cn (Z.B.); 2 Laboratory for Marine Fisheries Science and Food Production Processes, Qingdao National Laboratory for Marine Science and Technology, Qingdao 266237, China; 3 Laboratory of Tropical Marine Germplasm Resources and Breeding Engineering, Sanya Oceanographic Institution, Ocean University of China, Sanya 572000, China; 4 Weihai Changqing Ocean Science and Technology Co., Ltd., Weihai 264300, China; lulongfei567@163.com

**Keywords:** paralytic shellfish toxins, Zhikong scallop, gills, weighted gene co-expression network, *OCTN1*s

## Abstract

Bivalve molluscs are filter-feeding organisms that can accumulate paralytic shellfish toxins (PST) through ingesting toxic marine dinoflagellates. While the effects of PST accumulation upon the physiology of bivalves have been documented, the underlying molecular mechanism remains poorly understood. In this study, transcriptomic analysis was performed in the gills of Zhikong scallop (*Chlamys farreri*) after 1, 3, 5, 10, and 15 day(s) exposure of PST-producing dinoflagellate *Alexandrium minutum*. Higher numbers of differentially expressed genes (DEGs) were detected at day 1 (1538) and day 15 (989) than that at day 3 (77), day 5 (82), and day 10 (80) after exposure, and most of the DEGs were only regulated at day 1 or day 15, highlighting different response mechanisms of scallop to PST-producing dinoflagellate at different stages of exposure. Functional enrichment results suggested that PST exposure induced the alterations of nervous system development processes and the activation of xenobiotic metabolism and substance transport processes at the acute and chronic stages of exposure, respectively, while the immune functions were inhibited by PST and might ultimately cause the activation of apoptosis. Furthermore, a weighted gene co-expression network was constructed, and ten responsive modules for toxic algae exposure were identified, among which the yellow module was found to be significantly correlated with PST content. Most of the hub genes in the yellow module were annotated as solute carriers (*SLC*s) with eight being *OCTN1*s, implying their dominant roles in regulating PST accumulation in scallop gills. Overall, our results reveal the gene set responding to and involved in PST accumulation in scallop gills, which will deepen our understanding of the molecular mechanism of bivalve resistance to PST.

## 1. Introduction

Bivalve molluscs are filter-feeding organisms mainly consuming microalgae and can accumulate and concentrate various toxins during harmful algal blooms (HABs). Paralytic shellfish toxins (PST) produced by marine dinoflagellates, such as *Alexandrium* spp. and *Gymnodinium* spp., are the most widespread and among the most potent shellfish contaminating biotoxins [1]. PST are a group of neurotoxic alkaloids including saxitoxin (STX) and its naturally occurring analogues, which can block the conduction of nerve impulses by targeting voltage-gated sodium channels (Nav), resulting in neuromuscular paralysis and metabolic stress in vertebrates [2,3]. Unlike humans and other mammals, bivalves possess toxin-resistant amino acids in Nav protein, thus they are able to tolerate high concentrations of PST [2,4]. In addition, gene family analysis indicated that the genes related to detoxification, antioxidation, and immune stress, such as glutathione S-transferase (*GST*) [5], ATP-binding cassette transporter (*ABC*) [6], superoxide dismutase (*SOD*) [7], glutathione peroxidase (*GPx*) [8], and 70-kDa heat shock protein (*HSP70*) [9], were significantly altered in expression after exposure to PST-producing *Alexandrium*, implying the involvement of various biological processes in PST resistance.

By far, most of the studies on the molecular responses of bivalves to toxic algae focused on the main PST loading organ, digestive gland [10,11]. Previous works have determined that the accumulation and distribution of PST in bivalves is tissue/organ-specific [4]. The gills of bivalves, which possess a huge surface area and are involved in respiration and ciliary suspension feeding, could also accumulate a large amount of PST [12,13]. PST-producing dinoflagellate exposure could affect the physiological and cellular processes of gills in bivalves, such as higher mucus production, melanisation, hemocyte infiltration, DNA damage, and oxidative stress [10,14,15,16]. However, the molecular mechanisms of PST accumulation in bivalve gills and their transcriptional response to PST (or the PST-producing species) during exposure are poorly studied.

Scallops represent an economically important species for marine farming and are consumed worldwide. They are often used as a model species in various toxicology studies due to their filter-feeding habits and their rapid accumulation of toxins. During HABs, scallops could accumulate PST with higher concentrations and longer retention times compared with many other bivalve species, resulting in substantial economic losses in aquaculture and grave public health consequences [17]. In the present study, through transcriptomic analysis, the overall and dynamic molecular responses of Zhikong scallop (*Chlamys farreri*) gills to PST-producing dinoflagellate *A. minutum* exposure were revealed, and the key module and candidate gene set involved in PST accumulation was further identified by weighted gene co-expression network analysis (WGCNA). Our study represents the first comprehensive transcriptomic study on PST accumulation and responses in the gills of bivalves.

## 2. Results and Discussion

### 2.1. Low Similarity in Differentially Expressed Genes (DEGs) among Different Stages of A. minutum Exposure

**Figure 1:**
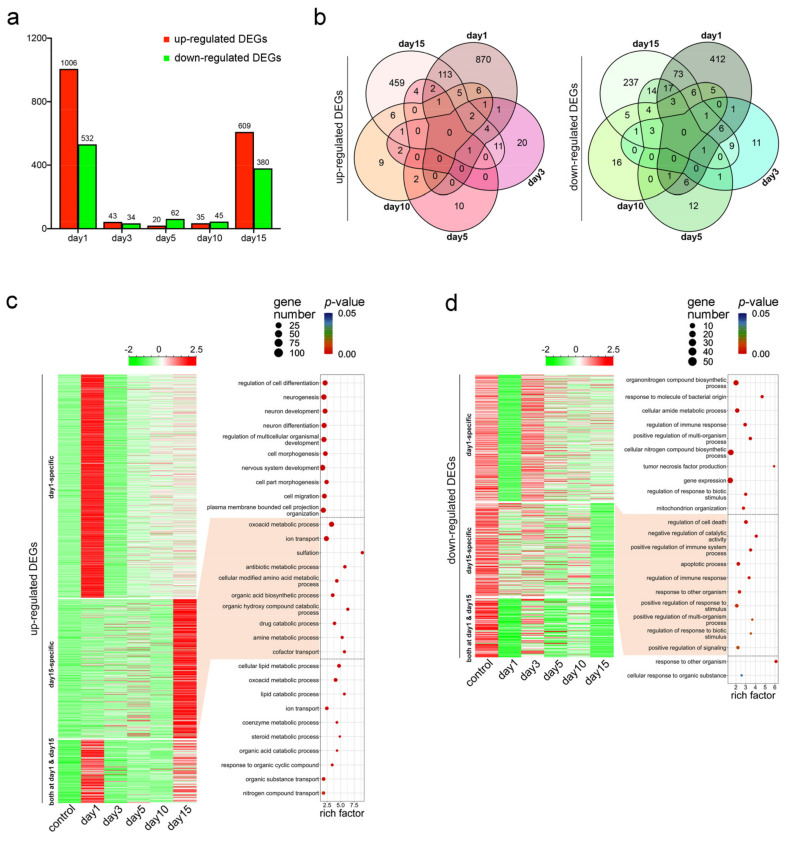
Differentially expressed genes (DEGs) in gills of *Chlamys farreri* after exposure to toxic *Alexandrium minutum*. (**a**) Comparison of the numbers of up- and down-regulated DEGs at each time point post-exposure. (**b**) Venn diagram of DEGs at different time points compared to the control group. Most up- and down-regulated DEGs were induced at 1 or 15 day(s) after exposure. (**c**,**d**) The heatmaps represented the expression patterns of up- and down-regulated DEGs specifically expressed at day 1 (day 1-specific), day 15 (day 15-specific), and co-expressed in both time points (both at day 1 and day 15), and the top 10 Gene Ontology (GO) terms in the biological process category of these genes.

In the current study, we analyzed the overall transcriptional changes in the gills of *C. farreri* exposed to *A. minutum* at five time points to identify the possible genes involved in PST response in this organ. A total of 1538, 77, 82, 80, and 989 DEGs (|log_2_FC(Fold Change)| > 1 and *p* < 0.05) were identified after 1, 3, 5, 10, and 15 day(s) exposure to *A. minutum*, respectively, when compared to the unexposed control (Figure 1a; Table S1). The number of DEGs at day 1 was the largest, with 1006 up-regulated genes and 532 down-regulated genes, followed by day 15, with 609 up-regulated and 380 down-regulated genes. At day 3, 5, and 10, the number of up-regulated genes was only 43, 20, and 35, respectively, and that of the down-regulated genes was 34, 62, and 45, respectively.

We previously detected PST profiles and concentrations in gills at each time point and found that gills were characterized by the domination of GTX2/3 and GTX1/4, which closely resembled that of the toxic algae fed to scallops [6], and also contained a small amount of the transformed types STX and NEO, which may be absorbed from the kidney, the main organ for PST transformation (Figure S1) [4]. The content of PST increased slightly during the first five days, then increased sharply and reached a peak at 15 days after exposure (Figure S1a) [4]. We observed the highest number of DEGs showing acute responses to toxic algae exposure at day 1. It has been reported that marine algal toxins cause more severe genotoxic and cytotoxic effects in bivalve cells at short exposition times [18]. The expression changes of a large number of DEGs might be caused by the toxic effects produced by acute exposure of toxic algae. From day 3 to day 10 of exposure, it is speculated to be a recovery period of scallop with a small number of genes being significantly altered in expression. After that, the strong response of gene expression was observed again at day 15 post-exposure, which was potentially related to the high accumulation of PST in scallop gills at this time point (Figure S1a), thus destroying the balance of gene expression. Further Venn analysis showed that most of the DEGs at day 1 and day 15 were time point-specific, that is, 870 (86.5%) up-regulated DEGs from day 1 were specifically induced just at day 1, and 459 (75.4%) DEGs from day 15 were specifically up-regulated just at day 15, while 412 (77.4%) and 237 (62.4%) DEGs were specifically down-regulated at day 1 and day 15 of exposure, respectively (Figure 1b). Only 113 up-regulated and 73 down-regulated DEGs were altered at both time points. These results indicate different response mechanisms in the gills of scallops to acute and chronic exposure to PST-producing algae. For the other three time points, the specifically up-regulated or down-regulated DEGs were no more than 50%. No DEG was significantly induced or inhibited at all five time points (Figure 1b).

### 2.2. Biological Processes of the Acute and Chronic Stages of Exposure

We further explored the function of up- and down-regulated DEGs through Gene Ontology (GO) enrichment analysis. The significantly enriched GO terms were only found at day 1 and day 15 post-exposure, and no enriched GO term was detected from the DEGs at day 3, 5, and 10 (Table S2).

As shown in Figure 1c, the up-regulated DEGs specifically identified at day 1 were mainly significantly enriched in the processes of nervous system development (e.g., neurogenesis, neuron development, and nervous system development). Previous studies have shown that the activity of STX at voltage-gated ion channels could have implications for neurodevelopment in vertebrates, such as inhibiting the normal outgrowth of neurite and inducing the alterations in neurogenesis [19,20]. Unlike the results in mammals, our data showed several genes function as the positive regulator of neurite outgrowth and neurogenesis, such as arylsulfatase B (*ARSB*) [21], neural-cadherin (*NCad*) [22], and serine/threonine-protein kinase (*unc-51*) [23], were up-regulated at day 1 after exposure, which may represent an acute response mechanism of scallop to protect the nervous system in gills from PST attack. By comparison, the up-regulated DEGs specifically detected at day 15 were mainly involved in the metabolic processes (e.g., drug metabolic process, oxoacid metabolic process, and antibiotic metabolic process) and substance transport processes (e.g., ion transport and cofactor transport) (Figure 1c). The DEGs involved in the metabolic process included the members of cytochrome P450 (*CYP450*s), ATP-binding transporter cassettes (*ABC*s), and sulfotransferases (*SULT*s), as well as the genes encoding oxidoreductases, such as peroxisomal sarcosine oxidase (*PIPOX*), aldehyde dehydrogenase (*ALDH2*), and sepiapterin reductase (*SPR*). *CYP450*s, *ABC*s, and *SULT*s are crucial members of detoxification [24], and the up-regulation of these genes after toxic algae exposure suggests the activation of inducible xenobiotic metabolism to detoxify PST in scallop gills. The DEGs involved in transport processes mainly belonged to solute carriers (SLCs), the transporter proteins that primarily mediate the absorption of small molecules into cells [25]. A large number of *SLC* genes were up-regulated at day 15, at which time point scallop also contained the highest PST, implying their possible involvement in PST transport or absorption. Besides, the up-regulated DEGs related to lipid metabolic-related processes (e.g., cellular lipid metabolic process and lipid catabolic process) were induced at both time points (Figure 1c), including peroxisomal acyl-coenzyme A oxidase 1 (*ACOX1*), peroxisomal 2,4-dienoyl-CoA reductase (*DECR2*), and peroxisomal multifunctional enzyme type 2 (*HSD17B4*). Garcia et al. reported that exposure to marine biotoxins can cause oxidative stress, and lead to the accumulation of reactive oxygen species (ROS), thus causing damage to lipids [26]. The expression changes of lipid metabolism-related genes in scallop gills may be related to oxidative stress caused by PST exposure.

On the other hand, the down-regulated DEGs specifically identified at day 1 were significantly enriched in immunological processes (e.g., response to molecule of bacterial origin, regulation of immune response, and regulation of response to biotic stimulus), while the DEGs exclusively found at day 15 were mainly enriched in the processes of apoptosis (e.g., regulation of cell death and apoptotic process) and immune responses (e.g., positive regulation of immune system process, regulation of immune response, and positive regulation of response to stimulus). The down-regulated DEGs shared at both time points were enriched in immune responses, including response to other organism and cellular response to organic substance (Figure 1d). The immune system of marine bivalves is sensitive to algal toxins. Chi et al. found that exposure to domoic acid impaired immune functions in the bay scallop [27]. Abi-Khalil et al. reported that exposure to PST-producing dinoflagellates significantly increased the susceptibility of oysters to pathogenic vibrio [28]. The down-regulation of immune-related pathways at both acute and chronic stages of exposure in scallop indicates PST may cause a negative impact on immune functions. Besides, our data also showed a down-regulation of genes related to apoptosis at day 15, including the genes encoding baculoviral IAP repeat-containing proteins (BIRCs) and caspases (CASPs). *BIRC*s are important negative regulators of apoptosis [29], thus the inhibition expression of *BIRC*s after toxic algae exposure may eventually activate apoptosis in scallop. Given that apoptosis is a mechanism to remove unwanted or damaged cells, the activation of this process seems to represent a protective mechanism for scallop to cope with damaged cells caused by PST accumulation.

### 2.3. Yellow Module of Gene Network Is Associated with PST Accumulation

**Figure 2:**
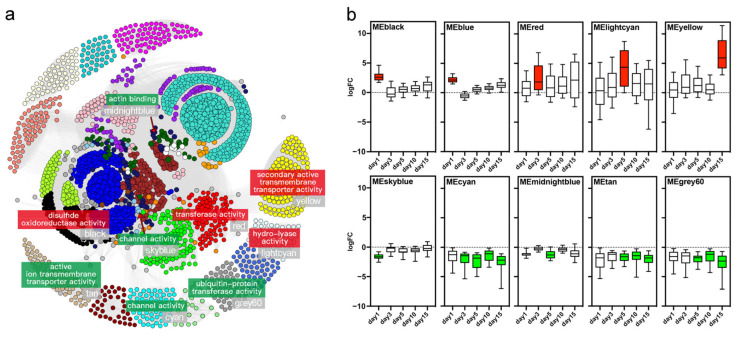
(**a**) Gene co-expression network in gills after exposure to *A. minutum*. For network visualization, each module is represented by a different color. Representative significant GO terms are shown for each responsive module. (**b**) The boxplot of DEGs for ten responsive modules based on the fold change of their expression levels.

A gene co-expression network was constructed from 18 transcriptome datasets to screen the toxin responsive modules. A total of 30 distinct modules were obtained from the expression data of 24,928 genes using a dynamic tree cutting algorithm, with module sizes ranging from 34 to 4773 (Figure 2a). Ten modules were identified as responsive modules, including five up-regulated and five down-regulated modules. Among them, MEblack, MEblue, MEred, MElightcyan, and MEyellow significantly enriched the up-regulated DEGs identified at day 1, 1, 3, 5, and 15 post-exposure, respectively. For down-regulated DEGs, MEskyblue, MEcyan, MEmidnightblue, MEtan, and MEgrey60 enriched the DEGs of 1 (day 1), 4 (day 3, 5, 10, 15), 1 (day 5), 3 (day 5, 10, 15), and 3 (day 5, 10, 15) time points of exposure, respectively (Figure 2b; Table S3). These responsive modules were found to be involved in diverse molecular functions, with up-regulated responsive modules mainly associated with transporter activity (e.g., secondary active transmembrane transporter activity) and various enzyme activity (e.g., disulfide oxidoreductase activity, hydro-lyase activity, and transferase activity), whereas down-regulated responsive modules primarily participated in channel activity, ubiquitin-protein transferase activity, and actin binding (Figure 2a).

**Figure 3:**
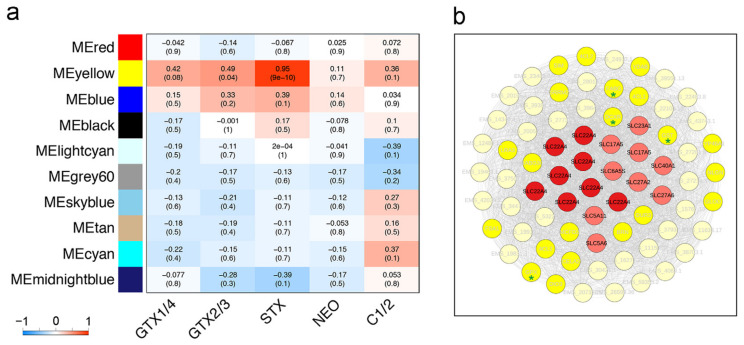
(**a**) Heatmap of module-trait relationships depicting correlations between module eigengenes and PST contents. Numbers in the table correspond to the correlation r and the *p* value in brackets. The degree of correlation is illustrated with the color legend. (**b**) Network visualization of hub genes in the yellow module (top 5% highest connectivity). Hub genes are connected by an edge if the topological overlap between them is more than 0.50. Each node represents a hub gene, which is labeled with gene name. Red nodes represent hub genes in SLC family, in which *SLC22*s are shown in dark red. The genes related to the oxidation-reduction process are marked with green asterisks.

As mentioned above, gills mostly accumulated the incoming toxins GTX1/4 and GTX2/3 from dinoflagellates, as well as a small amount of the transformed types STX, and the content of these toxins varied with the extension of exposure time (Figure S1a). To clarify the mechanism of toxin accumulation in scallop gills, we then analyzed the correlation between the expression patterns of each responsive module and PST contents. The hierarchical clustering indicated that the yellow module was significantly positively correlated with the content of PST, including STX (r = 0.95, *p* = 9e − 10) and GTX2/3 (r = 0.49, *p* = 0.04). The concentration of GTX1/4 also showed a positive correlation with the yellow module with the *p* value close to the significant level (r = 0.42, *p* = 0.08) (Figure 3a). These toxins were all associated with the yellow module, indicating that this module may be involved in the regulation of PST accumulation.

### 2.4. SLC Members Dominate the Hub Genes of the Yellow Module

As highly connected hub genes in a module play important roles in biological processes, hub gene analysis of the yellow module was conducted. A total of 38 annotated hub genes (top 5%) were identified in the yellow module (Figure 3b). Among them, 17 genes were *SLC* genes from seven subfamilies, including *SLC22* (8), *SLC5* (2), *SLC17* (2), *SLC27* (2), *SLC6* (1), *SLC23* (1), and *SLC40* (1). The expression of all these genes was positively correlated with the concentrations of STX (r = 0.9534~0.9579, *p* < 0.05), GTX2/3 (r = 0.4768~0.4902, *p* < 0.05), and GTX1/4 (r = 0.4164~0.4344, *p* = 0.07~0.08) (Table S4). The largest number of hub genes were from SLC22 subfamily. This subfamily comprises organic cation, zwitterion, and anion transporters (OCTs, OCTNs, and OATs), which mediate the transport of a variety of smaller and more hydrophilic substrates like various drugs and toxins [30]. Interestingly, the hub genes in SLC22 subfamily were all annotated as *SLC22A4* (*OCTN1*). In humans, OCTN1 could transport various cations into the cell across the plasma membrane [31]. Given that PST are a group of complex guanidine-based alkaloids with a cationic effect, our results indicate that the identified *OCTN1*s might function as the transporters for PST. Besides, several other *SLC* hub genes, such as *SLC5A6*, *SLC5A11*, *SLC6A5S*, and *SLC23A1*, came from the scallop-specifically expanded subfamilies, which belonged to the sodium-dependent transporters participating in the absorption and distribution of xenobiotics (*SLC5*) as well as neural signaling and homeostasis in scallops (*SLC6* and *SLC23*) [32,33,34,35]. Except for *SLC*s, the genes involved in the oxidation-reduction process, such as *GPX*, *DCXR*, *FABG*, and *GST*, were also identified as hub genes, indicating these genes may play a crucial role in protecting gill cells from oxidative stress caused by PST accumulation. Although functionally diverse hub genes have been identified, the high number of hub genes belong to SLC family, suggesting that *SLC*s, especially *OCTN1*s, might play a dominant role in regulating PST accumulation in gills.

## 3. Materials and Methods

### 3.1. PST-Producing Dinoflagellate A. minutum Exposure

Scallops were challenged previously by PST-producing dinoflagellate, *A. minutum* (AM-1 strain) [4]. Briefly, two-year-old adult *C. farreri* were collected and acclimated in filtered and aerated seawater at 12.5 ± 0.5 °C for three weeks by feeding the non-toxic algae, *Isochrysis galbana*. Then, scallop individuals were separated randomly into independent tanks with aeration. Before the exposure, *A. minutum* strain was cultivated independently and harvested at the late exponential growth phase [36,37]. Thereafter, 3 L of dinoflagellate cells were fed to each scallop once a day with a concentration of 2500 cells/mL. After feeding, scallops were sampled at 0 (control), 1 (acute exposure), 3, 5, 10, and 15 (chronic exposure) day(s) after exposure with three individuals at each time point. The gills were dissected, washed with sterile seawater, and immediately frozen at −80 °C.

### 3.2. Transcriptome Sequencing and DEGs Screening

Total RNA was isolated from the sampled gills following the protocol described by Hu et al. [38]. RNA integrity was checked with agarose gel electrophoresis, and the RNA concentration of each sample was measured using a Qubit RNA Assay Kit (Invitrogen). Eighteen RNA-seq libraries (three individuals for each time point) of the gills were constructed using the NEB Next mRNA Library Prep Kit, following the manufacturer’s instructions. The prepared libraries were subjected to paired-end 125 bp (PE125) sequencing on the Illumina HiSeq 2000 platform.

Raw reads were first cleaned by Trimmomatic and aligned on the genome of *C. farreri* using STAR aligner with default parameters [39,40]. Gene expression levels in terms of reads per kilobase of exon model per million mapped reads (RPKM) were calculated by HTseq and custom Perl scripts [41]. Differential gene expression analysis of each time point between the exposed and control groups was carried out using DESeq2 package, and genes with |log_2_FC| > 1 and *p* < 0.05 were defined as significant DEGs [42]. Venn diagrams were drawn to show the logical relations between the different sets of DEGs using the R package VennDiagram (version 1.6.16) [43]. To be aware of the function of DEGs, GO enrichment analysis was performed using TBtools [44]. Significantly enriched GO terms were defined by a hypergeometric test and a threshold of *p* value of less than 0.05.

### 3.3. Gene Co-Expression Network Analysis

Gene co-expression networks were constructed by means of the R package WGCNA using 18 transcriptomes from gills [45]. All the 24,928 genes expressed in gills were used for network construction, with the following parameters: softPower = 3, minimum module size = 30, and mergeCutHeight = 0.25. All gene modules corresponding to the branch cut-off of the gene tree were labeled in different colors. The hubness of a gene in the module was determined by intra-modular connectivity (Kwithin), and the top 5% of the genes with the highest connectivity in the module were defined as hub genes. GO enrichment analysis of each gene module was performed using TBtools. Cytoscape was used for the visualization of gene co-expression networks [46].

### 3.4. Identification of Key Module for Toxin Accumulation

To identify the responsive modules, an over-representation analysis of DEGs between toxin-exposed and control groups at each time point was performed for each module by a hypergeometric test (*p* < 0.01). Then, we further identified the correlation between these responsive modules and toxin contents. The eigengenes were first calculated using all genes in each responsive module, and then the eigengenes were associated with the contents of several PST, including carbamoyl toxins (GTX1/4, GTX2/3, STX, and NEO) and N-sulfocarbamoyl toxins C1/2. The only modules with *p* < 0.05 were considered as the key modules for toxin accumulation.

## 4. Conclusions

This study demonstrated the molecular response and toxin accumulation mechanism of scallop gills to PST exposure. Transcriptome analysis highlighted different response mechanisms of scallop to PST at the acute and chronic stages of exposure. The results suggested that PST exposure induced the alterations of nervous system development processes and the activation of xenobiotic metabolism and substance transport processes at the acute and chronic stages of exposure, respectively. PST also inhibited immune functions and ultimately caused the activation of apoptosis. Further gene network analysis detected a key module exhibiting significant correlations with the PST contents, the hub genes of which were mainly from the SLC family, especially the members encoding OCTN1s, indicating their dominant roles in regulating PST accumulation in gills. These findings provide candidate genes and biological processes for further studies on the molecular mechanisms of PST accumulation and responses in bivalves.

## Data Availability

Not applicable.

## Supplementary Materials

The following supporting information can be downloaded at: https://www.mdpi.com/article/10.3390/ijms23147912/s1.

## References

[1] Huss H.H., Smulders F.J.M., Collins J.D. (2002). Safety aspects associated with preharvest conditions of aquatic food products. Food Safety Assurance in the Pre-Harvest Phase.

[2] Bricelj V.M., Connell L., Konoki K., Macquarrie S.P., Scheuer T., Catterall W.A., Trainer V.L. (2005). Sodium channel mutation leading to saxitoxin resistance in clams increases risk of PSP. Nature.

[3] Llewellyn L.E. (2006). Saxitoxin, a toxic marine natural product that targets a multitude of receptors. Nat. Prod. Rep..

[4] Li Y., Sun X., Hu X., Xun X., Zhang J., Guo X., Jiao W., Zhang L., Liu W., Wang J. (2017). Scallop genome reveals molecular adaptations to semi-sessile life and neurotoxins. Nat. Commun..

[5] Lou J., Cheng J., Xun X., Li X., Li M., Zhang X., Li T., Bao Z., Hu X. (2020). Glutathione S-transferase genes in scallops and their diverse expression patterns after exposure to PST-producing dinoflagellates. Mar. Life Sci. Technol..

[6] Wang H., Liu S., Xun X., Li M., Lou J., Zhang Y., Shi J., Hu J., Bao Z., Hu X. (2021). Toxin- and species-dependent regulation of ATP-binding cassette (ABC) transporters in scallops after exposure to paralytic shellfish toxin-producing dinoflagellates. Aquat. Toxicol..

[7] Lian S., Zhao L., Xun X., Lou J., Li M., Li X., Wang S., Zhang L., Hu X., Bao Z. (2019). Genome-wide identification and characterization of *SOD*s in Zhikong scallop reveals gene expansion and regulation divergence after toxic dinoflagellate exposure. Mar. Drugs.

[8] Hlaing S.M.M., Lou J., Cheng J., Xun X., Li M., Lu W., Hu X., Bao Z. (2020). Tissue-biased and species-specific regulation of glutathione peroxidase (*GPx*) genes in scallops exposed to toxic dinoflagellates. Toxins.

[9] Hu B., Li M., Yu X., Xun X., Lu W., Li X., Li Y., Lou J., Wang S., Zhang L. (2019). Diverse expression regulation of *Hsp70* genes in scallops after exposure to toxic *Alexandrium* dinoflagellates. Chemosphere.

[10] Mat A.M., Haberkorn H., Bourdineaud J.P., Massabuau J.C., Tran D. (2013). Genetic and genotoxic impacts in the oyster *Crassostrea gigas* exposed to the harmful alga *Alexandrium minutum*. Aquat. Toxicol..

[11] Rolland J.L., Medhioub W., Vergnes A., Abi-Khalil C., Savar V., Abadie E., Masseret E., Amzil Z., Laabir M. (2014). A feedback mechanism to control apoptosis occurs in the digestive gland of the oyster *Crassostrea gigas* exposed to the paralytic shellfish toxins producer *Alexandrium catenella*. Mar. Drugs.

[12] De Oliveira David J.A., Salaroli R.B., Fontanetti C.S. (2008). Fine structure of *Mytella falcata* (Bivalvia) gill filaments. Micron.

[13] Liu Y., Kong F.Z., Xun X.G., Dai L., Geng H.X., Hu X.L., Yu R.C., Bao Z.M., Zhou M.J. (2020). Biokinetics and biotransformation of paralytic shellfish toxins in different tissues of Yesso scallops, *Patinopecten yessoensis*. Chemosphere.

[14] Braga A.C., Pereira V., Marçal R., Marques A., Guilherme S., Costa P.R., Pacheco M. (2020). DNA damage and oxidative stress responses of mussels *Mytilus galloprovincialis* to paralytic shellfish toxins under warming and acidification conditions—Elucidation on the organ-specificity. Aquat. Toxicol..

[15] Haberkorn H., Lambert C., Le Goïc N., Moal J., Suquet M., Guéguen M., Sunila I., Soudant P. (2010). Effects of *Alexandrium minutum* exposure on nutrition-related processes and reproductive output in oysters *Crassostrea gigas*. Harmful Algae.

[16] Borcier E., Morvezen R., Boudry P., Miner P., Charrier G., Laroche J., Hegaret H. (2017). Effects of bioactive extracellular compounds and paralytic shellfish toxins produced by *Alexandrium minutum* on growth and behaviour of juvenile great scallops *Pecten maximus*. Aquat. Toxicol..

[17] Tan K.S., Ransangan J. (2015). Factors influencing the toxicity, detoxification and biotransformation of paralytic shellfish toxins. Rev. Environ. Contam. Toxicol..

[18] Prego-Faraldo M.V., Valdiglesias V., Laffon B., Mendez J., Eirin-Lopez J.M. (2016). Early genotoxic and cytotoxic effects of the toxic dinoflagellate *Prorocentrum lima* in the mussel *Mytilus galloprovincialis*. Toxins.

[19] O’Neill K., Musgrave I.F., Humpage A. (2016). Low dose extended exposure to saxitoxin and its potential neurodevelopmental effects: A review. Environ. Toxicol. Pharmacol..

[20] O’Neill K., Musgrave I.F., Humpage A. (2017). Extended low-dose exposure to saxitoxin inhibits neurite outgrowth in model neuronal cells. Basic Clin. Pharmacol. Toxicol..

[21] Zhang X., Bhattacharyya S., Kusumo H., Goodlett C.R., Tobacman J.K., Guizzetti M. (2014). Arylsulfatase B modulates neurite outgrowth via astrocyte chondroitin-4-sulfate: Dysregulation by ethanol. Glia.

[22] Ribeiro M., Levay K., Yon B., Ayupe A.C., Salgueiro Y., Park K.K. (2020). Neural cadherin plays distinct roles for neuronal survival and axon growth under different regenerative conditions. eNeuro.

[23] Ogura K., Wicky C., Magnenat L., Tobler H., Mori I., Müller F., Ohshima Y. (1994). *Caenorhabditis elegans unc-51* gene required for axonal elongation encodes a novel serine/threonine kinase. Genes Dev..

[24] Livingstone D.R. (1991). Organic xenobiotic metabolism in marine invertebrates. Advances in Comparative and Environmental Physiology.

[25] Hong M. (2017). Biochemical studies on the structure-function relationship of major drug transporters in the ATP-binding cassette family and solute carrier family. Adv. Drug Del. Rev..

[26] Garcia C., Oyaneder J., Contreras H., Kovács A., Nagy P. (2017). Oxidative effects in aquatic organisms exposed to lipophilic marine biotoxins. Advances in Marine Biology.

[27] Chi C., Zhang C., Liu J., Zheng X. (2019). Effects of marine toxin domoic acid on innate immune responses in bay scallop *Argopecten irradians*. J. Mar. Sci. Eng..

[28] Abi-Khalil C., Lopez-Joven C., Abadie E., Savar V., Amzil Z., Laabir M., Rolland J.L. (2016). Exposure to the paralytic shellfish toxin producer *Alexandrium catenella* increases the susceptibility of the oyster *Crassostrea gigas* to pathogenic vibrios. Toxins.

[29] Silke J., Vaux D.L. (2001). Two kinds of BIR-containing protein—Inhibitors of apoptosis, or required for mitosis. J. Cell Sci..

[30] Roth M., Obaidat A., Hagenbuch B. (2012). OATPs, OATs and OCTs: The organic anion and cation transporters of the SLCO and SLC22A gene superfamilies. Br. J. Pharmacol..

[31] Pochini L., Galluccio M., Scalise M., Console L., Pappacoda G., Indiveri C. (2022). OCTN1: A widely studied but still enigmatic organic cation transporter linked to human pathology and drug interactions. Int. J. Mol. Sci..

[32] Xun X., Cheng J., Wang J., Li Y., Li X., Li M., Lou J., Kong Y., Bao Z., Hu X. (2020). Solute carriers in scallop genome: Gene expansion and expression regulation after exposure to toxic dinoflagellate. Chemosphere.

[33] Wright E.M., Turk E. (2004). The sodium/glucose cotransport family SLC5. Pflug. Arch..

[34] Bürzle M., Suzuki Y., Ackermann D., Miyazaki H., Maeda N., Clémençon B., Burrier R., Hediger M.A. (2013). The sodium-dependent ascorbic acid transporter family SLC23. Mol. Asp. Med..

[35] Pramod A.B., Foster J., Carvelli L., Henry L.K. (2013). SLC6 transporters: Structure, function, regulation, disease association and therapeutics. Mol. Asp. Med..

[36] Navarro J.M., Muñoz M.G., Contreras A.M. (2006). Temperature as a factor regulating growth and toxin content in the dinoflagellate *Alexandrium catenella*. Harmful Algae.

[37] García-Lagunas N., Romero-Geraldo R., Hernández-Saavedra N.Y. (2013). Genomics study of the exposure effect of *Gymnodinium catenatum*, a paralyzing toxin producer, on *Crassostrea giga*s’ defense system and detoxification genes. PLoS ONE.

[38] Hu X., Bao Z., Hu J., Shao M., Zhang L., Bi K., Zhan A., Huang X. (2006). Cloning and characterization of tryptophan 2,3-dioxygenase gene of Zhikong scallop *Chlamys farreri* (Jones and Preston 1904). Aquac. Res..

[39] Bolger A.M., Lohse M., Usadel B. (2014). Trimmomatic: A flexible trimmer for Illumina sequence data. Bioinformatics.

[40] Dobin A., Davis C.A., Schlesinger F., Drenkow J., Zaleski C., Jha S., Batut P., Chaisson M., Gingeras T.R. (2012). STAR: Ultrafast universal RNA-seq aligner. Bioinformatics.

[41] Anders S., Pyl P.T., Huber W. (2014). HTSeq-a Python framework to work with high-throughput sequencing data. Bioinformatics.

[42] Love M.I., Huber W., Anders S. (2014). Moderated estimation of fold change and dispersion for RNA-seq data with DESeq2. Genome Biol..

[43] Chen H., Boutros P.C. (2011). Venndiagram: A package for the generation of highly-customizable venn and euler diagrams in R. BMC Bioinform..

[44] Chen C., Chen H., Zhang Y., Thomas H.R., Frank M.H., He Y., Xia R. (2020). TBtools: An integrative toolkit developed for interactive analyses of big biological data. Mol. Plant..

[45] Langfelder P., Horvath S. (2008). WGCNA: An R package for weighted correlation network analysis. BMC Bioinform..

[46] Shannon P., Markiel A., Ozier O., Baliga N.S., Wang J.T., Ramage D., Amin N., Schwikowski B., Ideker T. (2003). Cytoscape: A software environment for integrated models of biomolecular interaction networks. Genome Res..

