# Supplementary figures and images for: Transcriptome and Network Analyses Reveal the Gene Set Involved in PST Accumulation and Responses to Toxic Alexandrium minutum Exposure in the Gills of Chlamys farreri
Source: Int J Mol Sci. 2022 Jul 18;23(14):7912. doi: 10.3390/ijms23147912 (PMC9324277; doi:10.3390/ijms23147912)

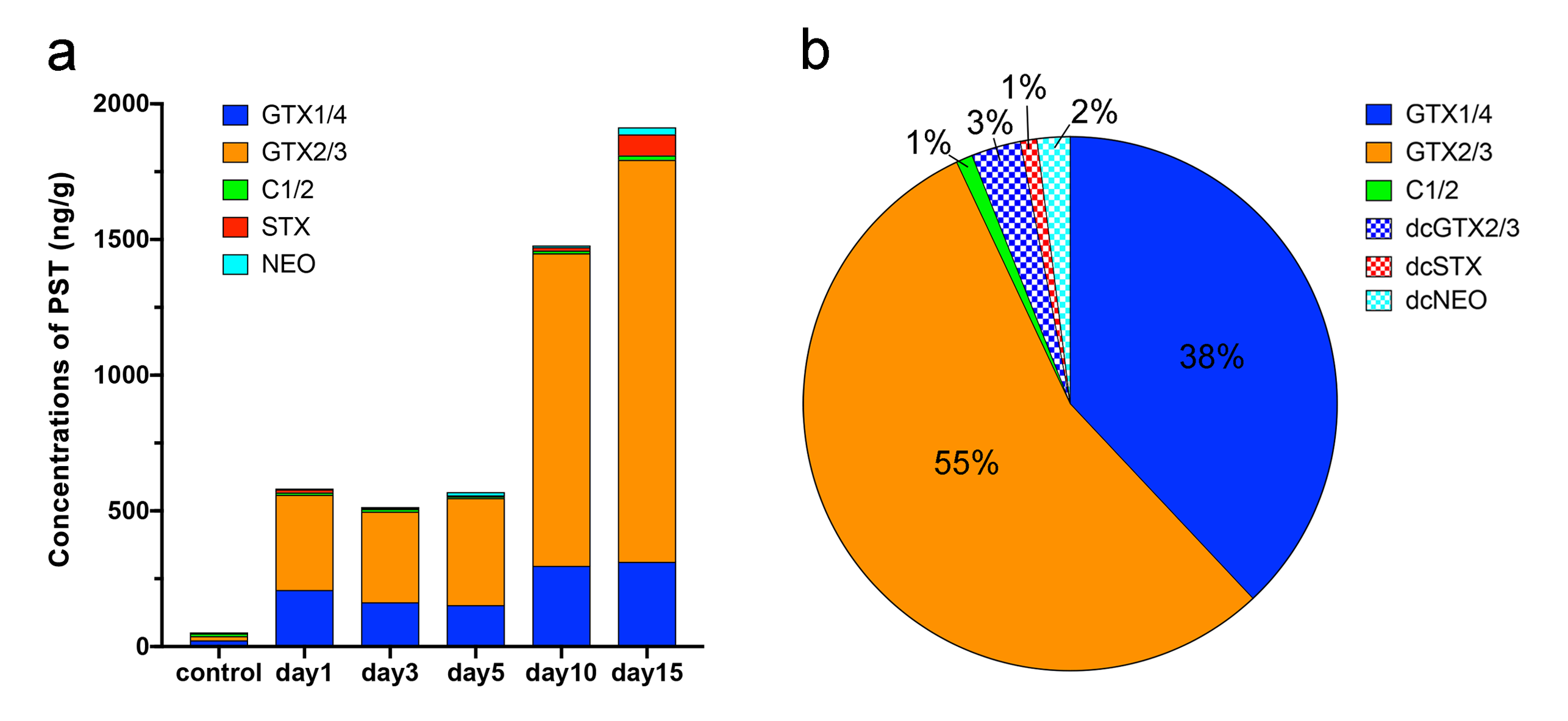

Supplement: Supplementary file 1 [file ijms-23-07912-s001.zip › Figure S1.tif]
